# Association of Inflammatory Responses and ECM Disorganization with HMGB1 Upregulation and NLRP3 Inflammasome Activation in the Injured Rotator Cuff Tendon

**DOI:** 10.1038/s41598-018-27250-2

**Published:** 2018-06-11

**Authors:** Finosh G. Thankam, Zachary K. Roesch, Matthew F. Dilisio, Mohamed M. Radwan, Anuradha Kovilam, R. Michael Gross, Devendra K. Agrawal

**Affiliations:** Departments of Clinical & Translational Science and Orthopedic Surgery, Creighton University School of Medicine, Omaha, NE 68178 USA

## Abstract

Inflammation and extracellular matrix (ECM) disorganization following the rotator cuff tendon injuries (RCTI) delay the repair and healing process and the molecular mechanisms underlying RCTI pathology are largely unknown. Here, we examined the role of HMGB1 and NLRP3 inflammasome pathway in the inflammation and ECM disorganization in RCTI. This hypothesis was tested in a tenotomy-RCTI rat model by transecting the RC tendon from the humerus. H&E and pentachrome staining revealed significant changes in the morphology, architecture and ECM organization in RC tendon tissues following RCTI when compared with contralateral control. Severity of the injury was high in the first two weeks with improvement in 3–4 weeks following RCTI, and this correlated with the healing response. The expression of proteins associated with increased HMGB-1 and upregulation of NLRP3 inflammasome pathway, TLR4, TLR2, TREM-1, RAGE, ASC, Caspase-1, and IL-1β, in the first two weeks following RCTI followed by decline in 3–4 weeks. These results suggest the association of inflammatory responses and ECM disorganization with HMGB1 upregulation and NLRP3 inflammasome activation in the RC tendons and could provide novel target(s) for development of better therapeutic strategies in the management of RCTI.

## Introduction

Sustained ECM disorganization with inflammation has been recognized as a major cause for the of pain and dysfunction in rotator cuff tendon injuries (RCTI)^1,2^. The underlying mechanisms leading to the disorganization of tendon matrix and activation of inflammatory pathways in otherwise hypovascular tissue like tendon is largely unknown. However, the upregulation of inflammatory cytokines and increased oxidative stress has been tightly associated with RCTI pathology which hurdles the healing responses. These cytokines are believed to be involved in the maintenance of tendon matrisome homeostasis by regulating the expression of extracellular matrix (ECM) genes, especially type 1 collagen by tenoblasts and tenocytes^3^. Moreover, the synovium has been considered to be a reserve for inflammatory cytokines as the level for IL-1β, IL-6 and other cytokines in the synovium correlates with the severity of RCTI. But, the influence of synovial cytokines in triggering RCTI pathology by aggravating ECM disorganization and inflammation are unknown^4^.

Evidence from RCTI patients and animal models suggest that the initial trigger for inflammation to be minor trauma of tendon (mostly unrecognized) which activates innate immune system through damage associated molecular patterns (DAMPs)^5^. ECM degradation products formed from collagen, fibronectin, hyaluronic acid and biglycans were proven to be potential DAMPs that upregulate pro-inflammatory mediators like IL-1β, TNF-α and IL-6 through NF-κB activation. These cytokines act as signal for MMP overexpression resulting in ECM degradation and inflammation not only in immune cells but also in non-immune cells like tenoblasts and tenocytes^6^^,^. ECM-derived DAMPs have also been reported to be the ligands for pattern recognition sequences like Toll Like Receptors (TLRs)^7^. Recently, we found the activation of intracellular DAMP, the HMGB1 (high mobility group box 1), in the shoulder tendon tissues of patients with massive RCTI suggesting the role of HMGB1 as an intracellular trigger for tendon inflammation^8^. However, the initial signal which triggers HMGB1 release is unknown.

HMGB1 (30kDda) is a DNA-binding protein which functions to maintain the integrity of nucleosome structure and gene regulation. Translocation of HMGB1 from nucleus to extracellular environment, either actively from immune cells or passively from necrotic non-immune cells, cast light to its potential role as a pro-inflammatory cytokine^9^. This was confirmed by treating human monocyte cell culture with recombinant HMBG1 that stimulated a school of inflammatory mediators like TNF-α, IL-1β, IL-1α, IL-8 and others^10^. Receptor for advanced glycation end products (RAGE) has been identified as a ligand for HMGB1, however, the role of HMGB1–RAGE axis in the pathogenesis of RCTI is not fully known. Apart from RAGE, activated HMGB1 also interacts with TLR4 which cumulate inflammatory responses especially by the activation of NLRP3 inflammasome^11,12^.

Inflammasomes are intracellular multi-protein assembly which is critical for eliciting inflammation by triggering the release of cytokines and is tightly associated with the pathophysiology of many diseases. For instance, the activation of Nucleotide-binding domain, Leucine-rich Repeat containing Protein-3 (NLRP3) inflammasome enhances the maturation and release of pro-inflammatory cytokines IL-1β and IL-18^13^. The recruitment of Apoptosis-associated Speck-like protein containing a CARD (ASC), the adapter protein, is initiated by NLRP3 receptor activation which ultimately leads to the proteolytic activation of pro-caspase-1 to caspase-1. Caspase-1 is inflammatory in nature which activates, matures and stabilizes IL-1β and IL-18 and initiates pyroptosis^14^. The priming signal for NLRP3 activation is induced by ligand binding to TLRs, especially TLR4 and TLR2, where the TLRs-NF-κB signaling enhances the expression of NLRP3 in the damaged cells. Being a ligand for TLR4, TLR2 and RAGE, HMGB1 can elicit synergistic effect on NLRP3-mediated inflammation which results in inflammation-associated events like membrane pore formation, K^+^ efflux, lysosomal rupture, mitochondrial dysfunction and oxidative stress^14,15^. IL-1β, the end product of NLRP3 pathway, mediates collagen matrix degradation by activating MMPs in connective tissues like bones and cartilages^16,17^. But, such reports are considerably rare when tendon tissues are concerned. The RC tendon tissue is hypovascular and is adapted to anaerobic environments^18^. However, extreme hypoxia following the RCTI triggers the release of HMGB1 and the downstream signaling resulting in IL-1β secretion^19^. Even though the presence of HMGB1, RAGE, and TLR4 have been reported in tendon tissues, there is no information on the existence, assembly, activation and execution of NLRP3-mediated inflammation in RCTI^18,19^. Here, we examined if the elevated level of HMGB1 is associated with the activation of NLRP3 inflammasome pathway in RC tendon tissues, and this could be the major cause of persistence inflammation and ECM disorganization following the RCTI.

## Results

### Induction of RCTI in rats

Macroscopic examination revealed the formation of disorganized regenerative scar tissue (neo-fibrous tissue) that was found to bridge the gap between the marked tendon stump and original osseous attachment of the RC tendon in all specimens. This tissue was then harvested for further analysis and compared to the contralateral control tendon. Approximately 4 mm gap between the marking suture and bone-bridge by the regenerative scar tissue was found in all specimens at all time-points. This indicates that the tendon was not able to anatomically heal to its native attachment on the greater tuberosity, but instead newly regenerated tissue was formed to bridge the gap between the transected tendon and bone. This regenerative tissue was the subject of our tissue analysis. The macroscopic anatomy of the RCTI tendon tissue showed remarkable difference in the appearance. Neo-fibrous tendon formation was present in the RCTI groups, as evidenced by the greyish white appearance, fragile nature, disorganization and loose texture. The control tendons displayed white shiny appearance and firm texture (Supplementary Figure 1). Neo-fibrous tissue formation was observed around 3–5 days and healing process progressed after 10–12 days and nearly normal organization of the RC tendon was observed around 22–24 days. However, the neo-tendon formed after 22 days following RCTI was found to be less glossy, distorted, and irregular while the control tendons were highly organized and white shiny in appearance. These findings support a time-dependent visual change in the physical appearance of RC tendon after RCTI.

### Histomorphology

**Figure 1:**
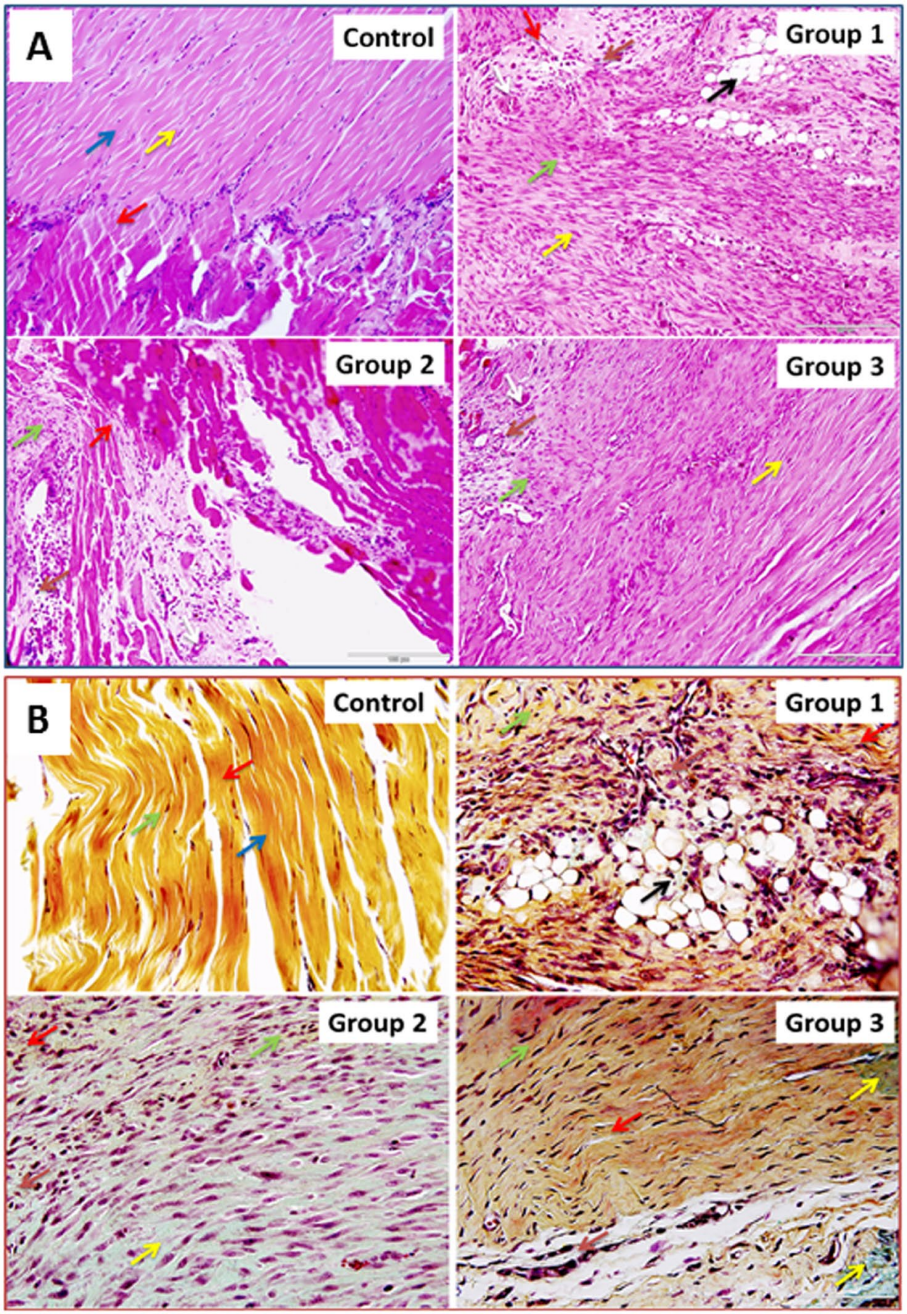
The histomorphological evaluation by H&E staining and Movat’s Pentachrome staining: The evaluations were performed in three groups and corresponding contralateral control. Group 1 involves RCTI tendons and contralateral control tendons of rats sacrificed 3–5 days post-injury, Group 2 involved the rats sacrificed 10–12 days post-injury and Group 3 consisted of RCTI tendons and contralateral control tendons sacrificed 22–24 days after injury. (**A**) H&E staining for rat RC tendons showing the status of ECM organization/disorganization following RCTI and healing in the Control, Group-1, Group-2 and Group-3. Yellow arrows show tenocytes, blue arrows point intact ECM, green arrows indicate ECM disorganization, red arrows show muscle-tendon interphase, black arrow points fatty infiltration, white arrows indicate angiogenesis and brown arrows show inflammation (due to the presence of neutrophils and monocytes). The control group image is the representative of 21 normal contralateral shoulder tendon in all three groups and in the RCTI group, each image represents 7 RCTI tendons. The images were acquired in 20x magnification. (**B**) Pentachrome staining for rat RC tendons showing ECM organization/disorganization and composition following RCTI groups and in the control group. Yellow arrows show collagen, blue arrows point intact ECM, green arrows indicate ECM disorganization, red arrows show muscle tissue, black arrow points fatty infiltration, green arrows indicate tenocytes and brown arrows reveal inflammation. The control group image represents 21 normal contralateral shoulder tendon and the other groups (1, 2 and 3) also represent 7 RCTI tendons. The images were acquired in 40x magnification.

The H&E staining revealed a considerable alteration in morphology, architecture and ECM organization in RC tendon tissues following RCTI when compared with control (Fig. 1A). The high field view of the RCTI pathology is shown in Supplementary video 1. The ECM disorganization was high on 3–5 days (Group-1) and 10–12 days (Group-2) post-injury, and milder ECM disorganization was observed on 22–24 days post-injury (Group-3). The qualitative microscopic evaluations revealed an increased cellular density in Group-1 and Group-2 RCTI tendons and were found to be decreased in Group-3 during the course of time. The tenocytes with their elongated nuclei were evident in intact control tendon, but in the RCTI tendons the tenocytes showed mostly oval nuclei and localized proliferation. The tendon tissue of six out of seven RCTI rats in Group-1 showed the presence of fatty infiltration which was minimal in Group-2 (2 out of 7) while completely absent in the RCTI tendons of Group-3. Vasculature was also observed in all groups. The presence of leukocytes was observed in all groups which were identified by their characteristic nuclei. The localized inflammation in the RCTI tendons were evident by the presence of neutrophils (segmented nucleus), and monocytes (kidney-shaped nucleus).

Qualitative examination of pentachrome staining revealed disorganization of collagen in the tendon tissues of RCTI rats compared to controls. The yellow staining for collagen was minimal in the Group-1 and Group-2; but in Group-3 partial reorganization of collagen was evident. The collagen disorganization was found to be increased after day three, peaked after day 10 and improved after day 22 in RCTI tendons of Group-1 and Group-2, but did not completely restore as in the control. Fatty infiltration and inflammation (characterized by the colonization of leukocytes and tenoblast cells in the vicinity of disorganized ECM) were observed, and the results were consistent with those found in the H&E staining. The deposition of ground substance (mucins) in the tendon was also found to be maximum in Group-2 and minimum in Group-3. The major changes in ECM composition associated with RCTI are shown in Fig. 1B. The high power view of the RCTI pathology is given in Supplementary video 2.

### Expression of Protein Mediators

**Figure 2:**
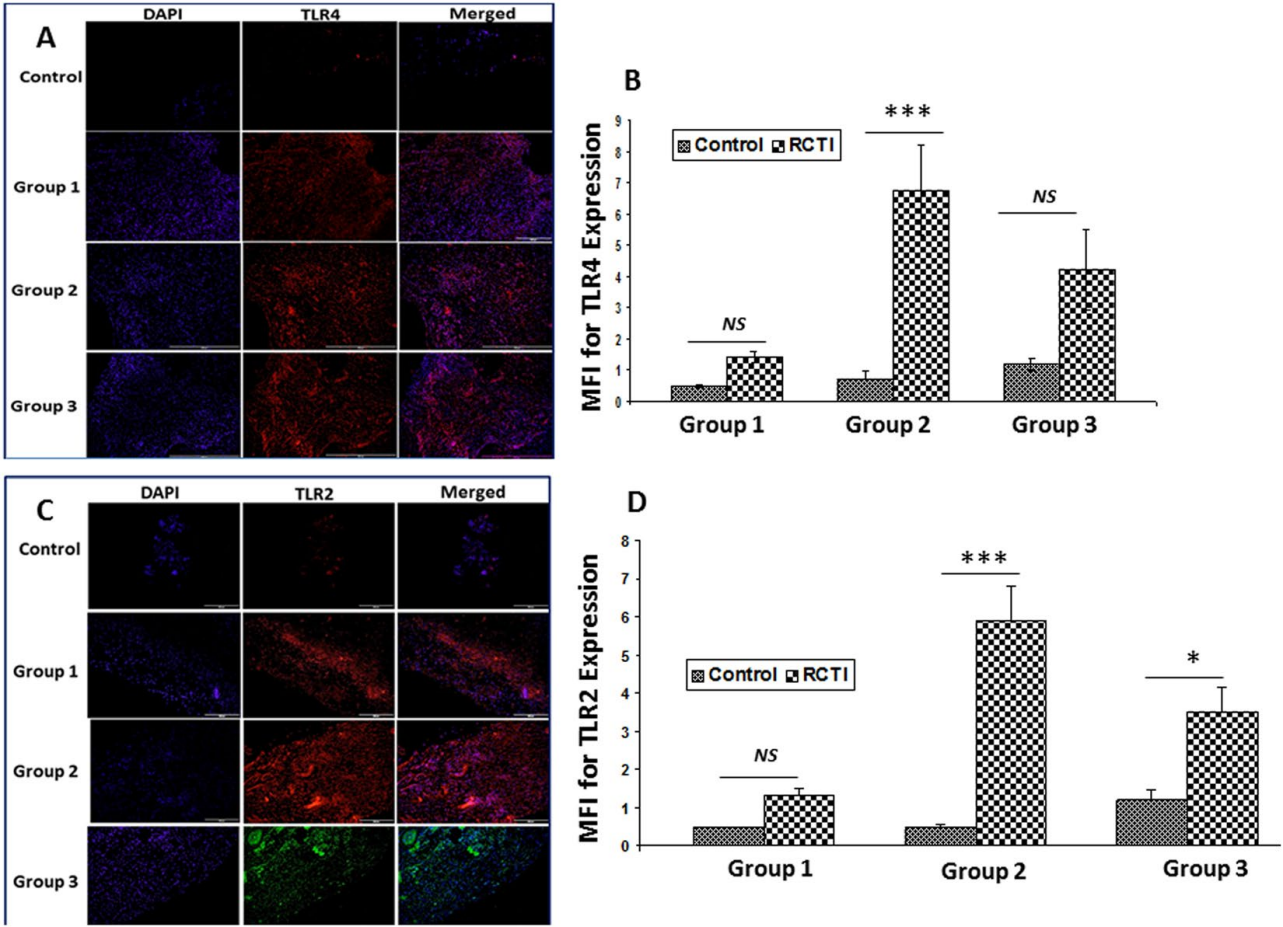
Immunofluorescence analysis for the expression of (**A**) TLR4 and (**C**) TLR2 showing increased expression in RCTI group in comparison to control. Images in the top row are histological sections of control group, and images in the bottom rows are histological sections of RCTI tendons harvested at 3–5 days (Group-1), 10–12 days (Group-2) and 22–24 days (Group-3). Images in the left column show nuclear staining with DAPI; the images in the middle column show expression of TLR4/TLR2 while the images in the right column show overlay of TLR4/TLR2 staining with DAPI. Images were acquired at 20x magnification using CCD camera attached to the Olympus microscope. (**B** and **D**) The image shows quantification of protein expression. The intensity of protein expression as observed through immunofluorescence was acquired and the mean fluorescence intensity (MFI) was quantified from each contralateral control and RCTI specimen. The graphs represent MFI mean values with standard error. (NS – non-significant, *P < 0.05, **P < 0.01 and ***p < *0.001*).

**Figure 3:**
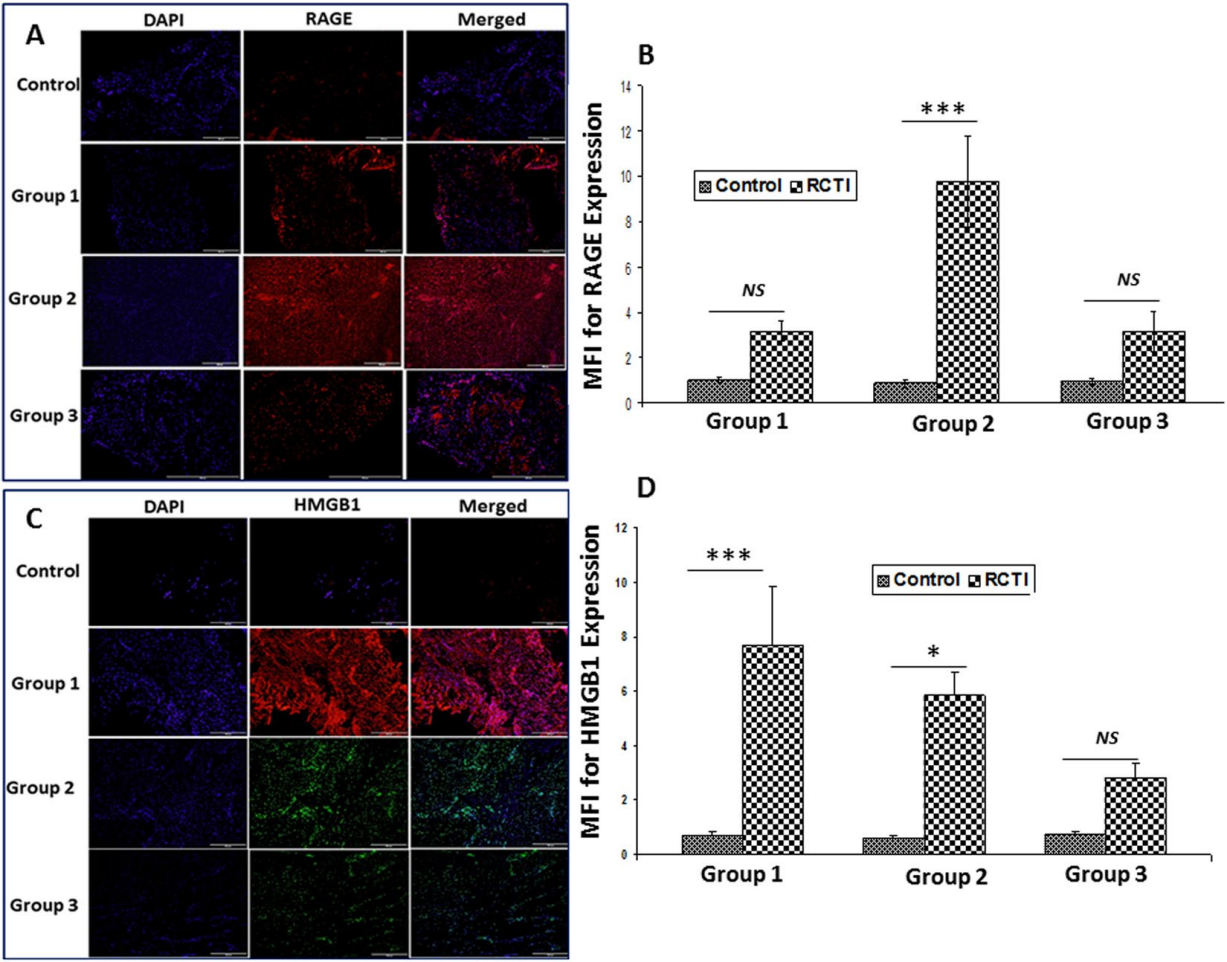
Immunofluorescence analysis for the expression of (**A**) RAGE and (**C**) HMGB1 showing increased expression in RCTI group compared to control. Images in the top row are histological sections of control group, and images in the bottom rows are histological sections of RCTI tendons harvested at 3–5 days (Group-1), 10–12 days (Group-2) and 22–24 days (Group-3). Images in the left column show nuclear staining with DAPI; the images in the middle column show expression of RAGE/HMGB1 while the images in the right column show overlay of RAGE/HMGB1 staining with DAPI. Images were acquired at 20x magnification using CCD camera attached to the Olympus microscope. The image shows quantification of the expression of RAGE (**B**) and HMGB1 (**D**). The intensity of gene expression as observed through immunofluorescence was acquired and the mean fluorescence intensity (MFI) was quantified from each contralateral control and RCTI specimen. The graphs represent MFI mean values with standard error. (NS – non-significant, *P < 0.05, **P < 0.01 and ***p < *0.001*).

The average cell count was found to be approximately 220, 530, 451 and 408 in the control, RCTI Group-1, RCTI Group-2 and RCTI Group-3, respectively. This corresponds to approximately 240%, 204% and 185% increase in cell density in the RCTI Group-1, RCTI Group-2 and RCTI Group-3, respectively, compared to the contralateral controls. Immunofluorescence analysis for the expression of proteins associated with NLRP3 inflammasome pathway revealed their considerable alterations in RCTI rats with respect to the healing response when compared to the controls. TLR4, the receptor molecule for NLRP3 inflammasome assembly, was found to be increased after 3 days (Group-1), peaked at 10–12 days (Group-2) and returned to nearly normal after 22–24 days (Group-3) (Fig. 2A and B) in the RCTI groups. Interestingly, TLR2 also showed similar trend (Fig. 2C and D). RAGE, another potent receptor for inflammasome pathway, also revealed a steep increase in the expression in Group-2 tendons of RCTI rats and then declined in Group-3 to the levels similar to those in Group-1 (Fig. 3A and B). Also, the expression of DAMP molecule HMGB1 was increased in the tendons following RC injury in both Group-1 and Group-2 and declined in Group-3. (Fig. 3C and D).

**Figure 4:**
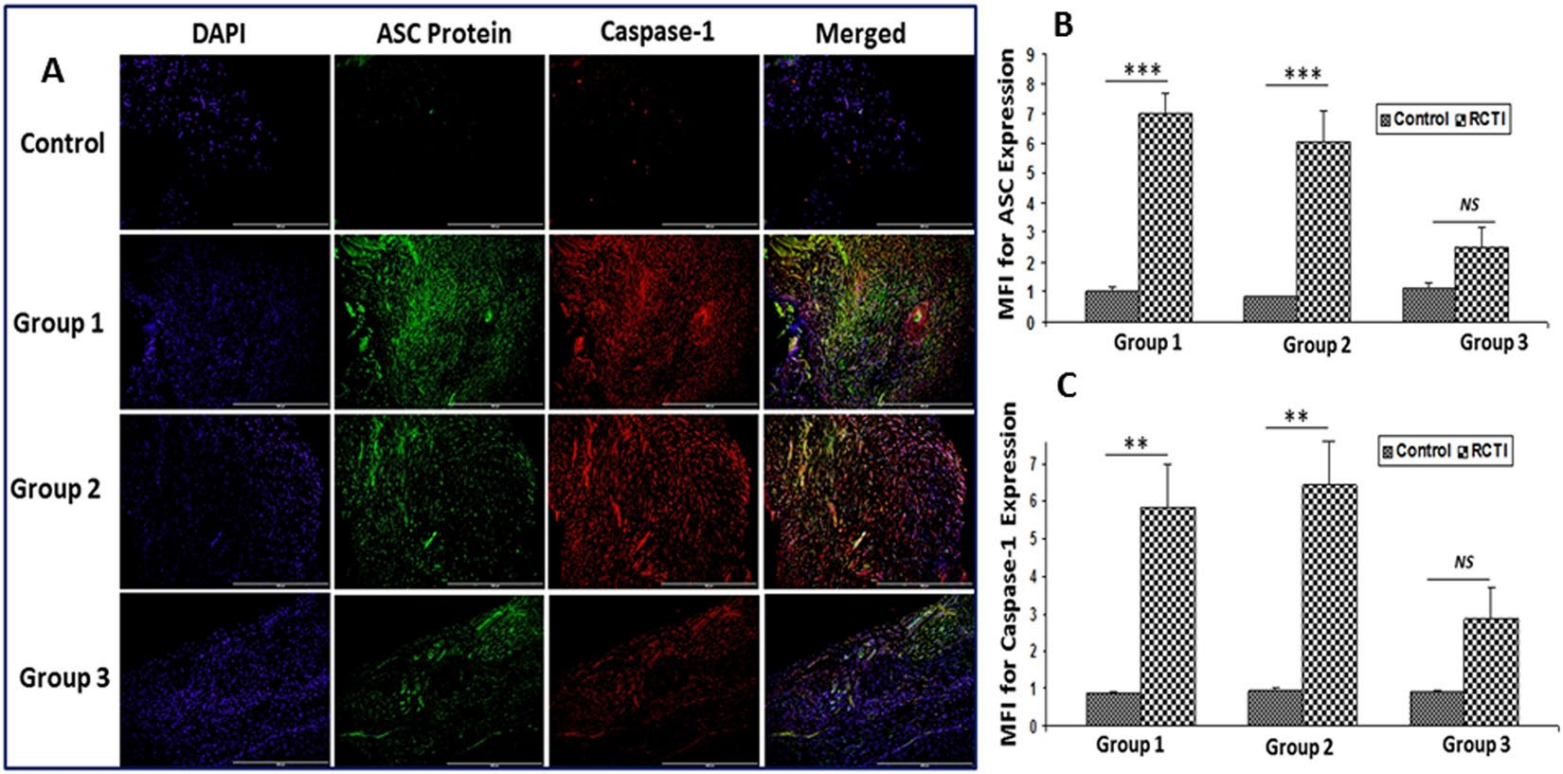
(**A**) Immunofluorescence analysis for the expression of ASC protein and Caspase-1 showing increased expression in RCTI group on comparison with control. Images in the top row are histological sections of control group, and images in the bottom rows are histological sections of RCTI tendons harvested at 3–5 days (Group-1), 10–12 days (Group-2) and 22–24 days (Group-3). Images in the left column show nuclear staining with DAPI; the images in the middle column show expression of ASC protein and Caspase-1 while the images in the right column show overlay of ASC protein and Caspase-1 staining with DAPI. Images were acquired at 20x magnification using CCD camera attached to the Olympus microscope. The image shows quantification of the expression of ASC protein (**B**) and Caspase-1 (C). The intensity of gene expression as observed through immunofluorescence was acquired and the mean fluorescence intensity (MFI) was quantified from each contralateral control and RCTI specimen. The graphs represent MFI mean values with standard error. (NS – non-significant, *P < 0.05, **P < 0.01 and ***p < *0.001*).

**Figure 5:**
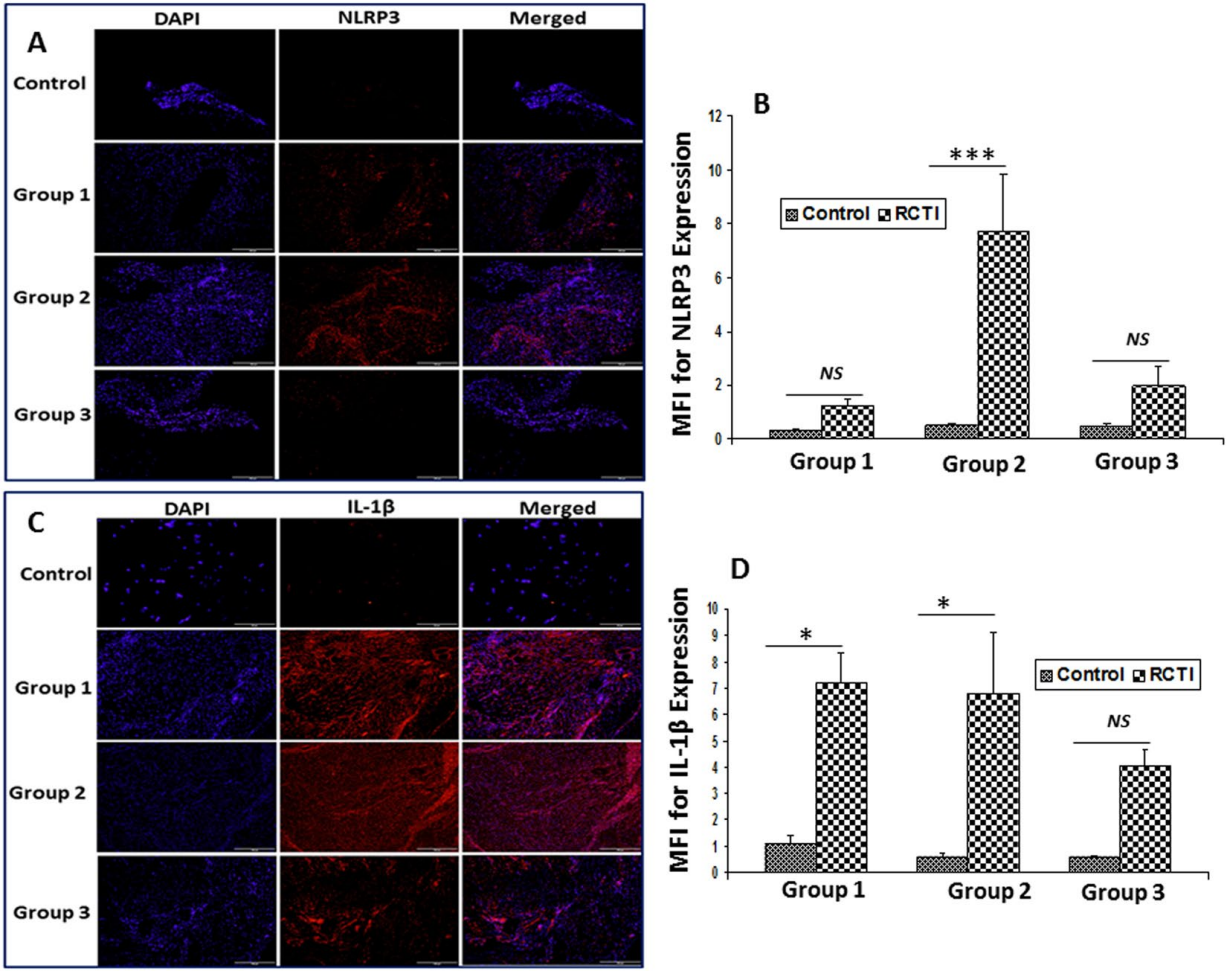
Immunofluorescence analysis for the expression of (**A**) NLRP3 and (**C**) IL-1β showing increased expression in RCTI group on comparison with control. Images in the top row are histological sections of control group, and images in the bottom rows are histological sections of RCTI tendons harvested at 3–5 days (Group-1), 10–12 days (Group-2) and 22–24 days (Group-3). Images in the left column show nuclear staining with DAPI; the images in the middle column show expression of NLRP3/IL-1β while the images in the right column show overlay of NLRP3/IL-1β staining with DAPI. Images were acquired at 20x magnification using CCD camera attached to the Olympus microscope. The image shows quantification of the expression NLRP3 (**B**) and IL-1β (**D**). The intensity of gene expression as observed through immunofluorescence was acquired and the mean fluorescence intensity (MFI) was quantified from each contralateral control and RCTI specimen. The graphs represent MFI mean values with standard error. (NS – non-significant, *P < *0.05*, **P < 0.01 and ***p < *0.001*).

ASC, the adaptor protein for inflammasome assembly, showed a progressive downregulation in the tendons following RC injury in Group-1 to Group-3 rats; these findings correlated with the healing status of the animals (Fig. 4A and B). Caspase-1 expression was upregulated in Group-1 and Group-2 and considerably decreased in Group-3 (Fig. 4A and C). The NLRP3 expression was increased in the tendons of Group-1 to Group-2, but the NLRP3 expression in the tendons of Group-3 rats was variable with less expression compared to Group-1, but more expression than in Group-2 (Fig. 5A and B). Similar trend was observed for the downstream mediator IL-1β (Fig. 5C and D). Moreover, the contralateral control tendons displayed minimal expression for all mediators which was considered to be basal level (Figs 2–5**)**. Thus, these findings show that the mediators of NLRP3 inflammasome peak around 10–12 days following tendon injury (Group-2) which declines after 22–24 days.

**Figure 6:**
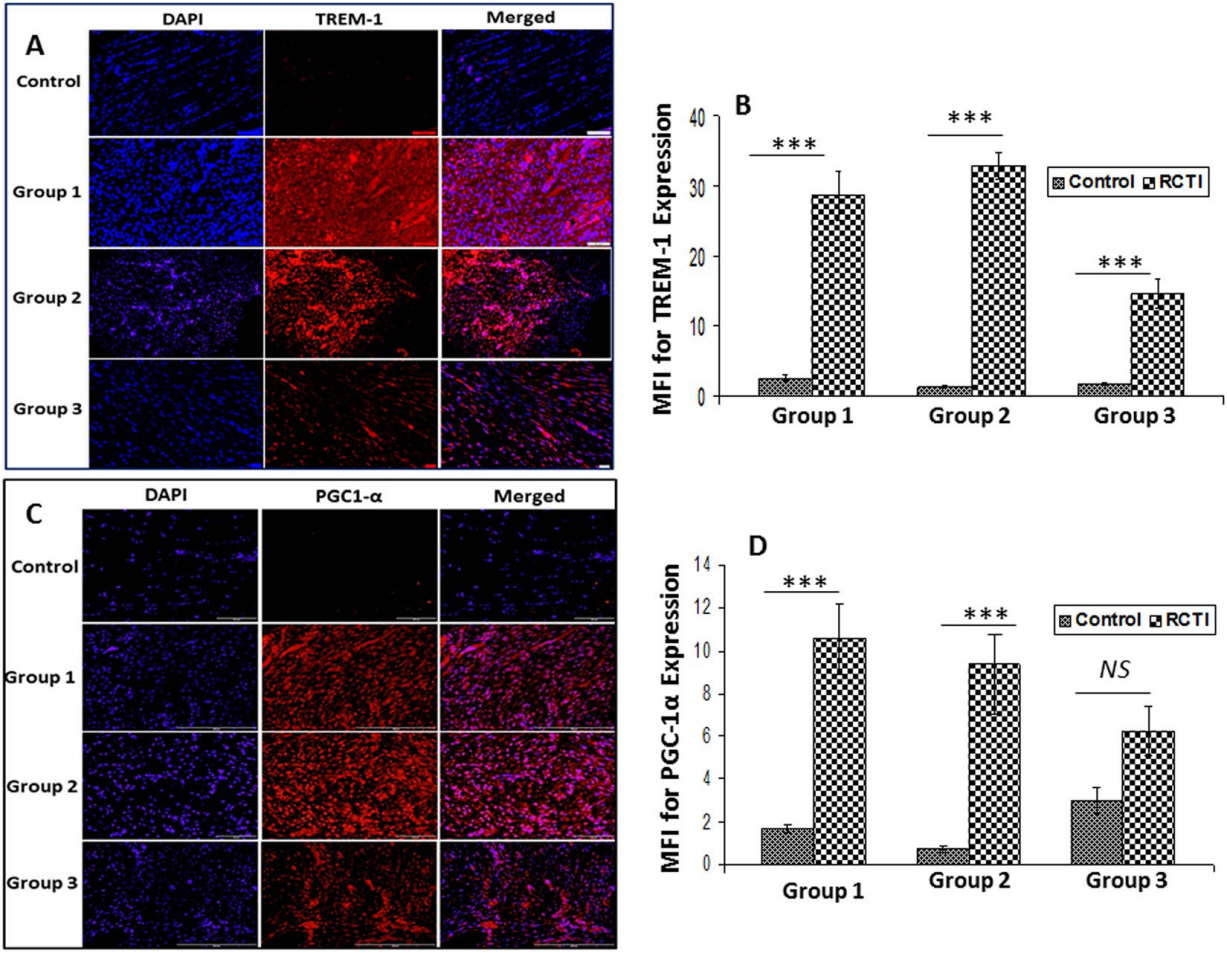
Immunofluorescence analysis for the expression of (**A**) TREM-1 and (**C**) PGC-1α showing increased expression in RCTI group on comparison with control. Images in the top row are histological sections of control group, and images in the bottom rows are histological sections of RCTI tendons harvested at 3–5 days (Group-1), 10–12 days (Group-2) and 22–24 days (Group-3). Images in the left column show nuclear staining with DAPI; the images in the middle column show expression of TREM-1/PGC-1α while the images in the right column show overlay of TREM-1/PGC-1α staining with DAPI. Images were acquired at 20x magnification using CCD camera attached to the Olympus microscope. The image shows quantification of the expression TREM-1 (**B**) and PGC-1α (**D**). The intensity of gene expression as observed through immunofluorescence was acquired and the mean fluorescence intensity (MFI) was quantified from each contralateral control and RCTI specimen. The graphs represent MFI mean values with standard error. (NS – non-significant, *P < 0.05, **P < 0.01 and ***p < *0.001*).

**Figure 7:**
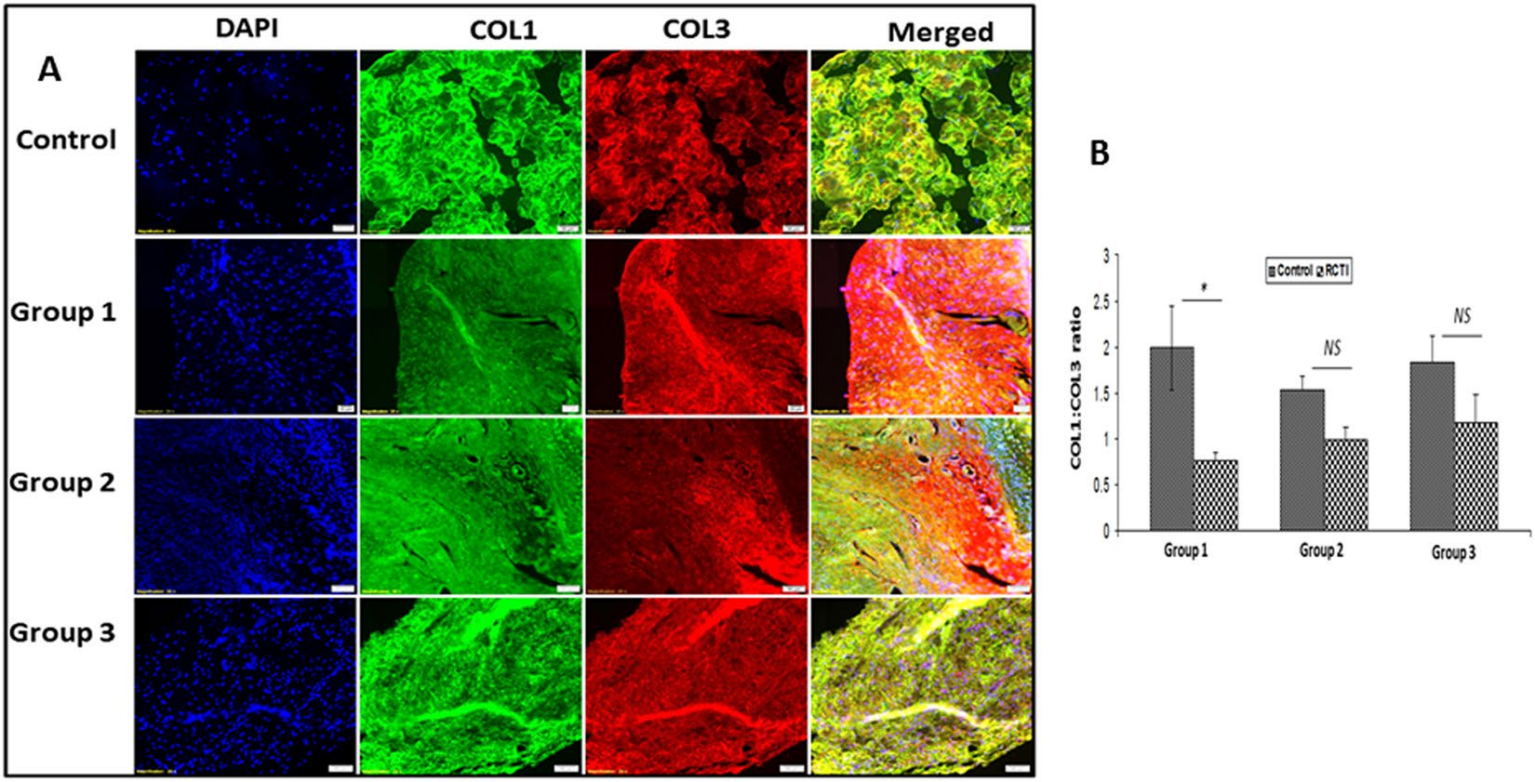
(**A**) Immunofluorescence analysis for the expression of COL1 (green) and COL3 (red) showing increased expression in RCTI group on comparison with control. Images in the top row are histological sections of control group, and images in the bottom rows are histological sections of RCTI tendons harvested at 3–5 days (Group-1), 10–12 days (Group-2) and 22–24 days (Group-3). Images in the left column show nuclear staining with DAPI; the images in the middle column show expression of COL1 and COL3 while the images in the right column show overlay of COL1 and COL3 staining with DAPI. Images were acquired at 20x magnification using CCD camera attached to the Olympus microscope. (**B**) The image shows quantification of the expression of COL1 and COL3. The intensity of gene expression as observed through immunofluorescence was acquired and the mean fluorescence intensity (MFI) was quantified from each contralateral control and RCTI specimen. The MFI of same fields were used to calculate the ratio of COL1:COL3. The graphs represent mean values of COL1:COL3 ratio with standard error. (NS – non-significant, *P < 0.05, **P < 0.01 and ***p *<* *0.001*).

TREM-1 expression was found to be significantly elevated in the RCTI tendons of all three experimental groups following injury when compared with the corresponding contralateral controls. High expression of TREM-1 was observed in Group-2 RCTI tendons which was decreased in Group-3 (Fig. 6A and B). The expression of PGC-1α, a biomarker for mitochondrial biogenesis, was higher in Group-1 and Group-2 RCTI tendons compared to controls and that in Group-3, where it was not significantly different from the control group (Fig. 6C and D). The expression of tendon matrix proteins COL1 and COL3 was assessed and the results are expressed as the ratio of COL1-to-COL3. The MFI values corresponding to COL1 and COL3 from the same field were used for calculating the ratio. The ratio was found to be significantly reduced in Group-1 RCTI tendons when compared to the contralateral control. A progressive increase in COL1-to-COL3 ratio was observed in the RCTI tendons of Group-2 and Group-3, but the COL1:COL3 ratio in the RCTI tendons was significantly lower than their corresponding contralateral controls (Fig. 7A and B).

## Discussion

Factors other than age like diabetes, smoking, and infections in the late stage sustain inflammation and pain in the shoulder that adversely affect repair processes and healing rate, resulting in greater chance of reoccurrence^20^. The tendon healing proceeds through an inflammatory phase followed by proliferative phase, which, in turn, is followed by a remodeling phase. The inflammatory phase results in enhanced vascular permeability, neoangiogenesis and ultimately the recruitment of inflammatory cells to the target site. The inflammatory cell activation increases the local concentration of cytokines and growth factors which trigger the migration and maturation of macrophages and tenoblast proliferation. This proliferative phase paves way to remodeling phase which is characterized by deposition, organization, crosslinking and maturation of collagen fibers in tendon ECM^21^. The persistence of inflammation at the injury site sustains ECM disorganization and delays the healing responses.

The focus of this study was to examine the ECM disorganization and inflammation in regard to altered collagen I to III ratio and the expression of the mediators of NLRP3 inflammasome in the shoulder tendon tissue of tenotomy-RCTI rat model. The tenotomy model is beneficial to evaluate the RCTI injury mechanisms and self-healing responses^22^ since the chronic tendon damage in rats can be healed in 2–4 months^21^. Moreover, the healing responses are active in the younger rats^21^. The current study used contralateral side as a control to compare healing effects for a period of 24 days. The extent of ECM disorganization was found to be reduced during the course of time with an increased collagen I to III ratio with a concomitant decrease in inflammatory markers (Fig. 7A and B). However, the underlying molecular mechanisms leading to the activation of NLRP3 inflammasome and its association with ECM disorganization warrant further research.

The findings in our animal model revealed a consistent expression of HMGB1 in the RC tendon tissues throughout the period of 24 days following injury. The HMGB1 is released to extracellular spaces as all-thiol state which acts on the Ig superfamily receptor RAGE to elicit inflammatory responses. Also, under oxidative environment the Cys-23 and Cys-45 of HMGB1 form disulfide linkage and the HMGB1-disulfide interacts with TLR4^23^. However, the Cys-106 of HMGB1 should be maintained in –SH (thiol) state for activating TLR4^24^. The binding of HMGB1 to RAGE and TLR4 triggers the cytokine-inducing activities and subsequent inflammation^23^. In addition, HMGB1 may also activate TLR2 signaling^25^. The hypoxia associated with RCTI is a well-known trigger for oxidative stress, which could be a major cause for delayed healing response following RCTI^19,26^. Increased levels of HIF1α in the RCTI rats tendons revealed a hypoxic and ischemic environment at the vicinity of injury (data not shown in this article). These results signify the role of HMGB1 as the initial trigger for inflammation associated with RCTI and the increased hypoxic and oxidative stress following pathology cause the persistence of HMGB1, which, in turn, maintains RAGE, TLR4 and TLR2 in the activated state.

The inflammatory cytokine, IL-1β, was found to be tightly associated with the persistence of RCTI symptoms, similar to the initial trigger (HMGB1). The upregulation of IL-1β resulting from TLR2 and TLR4 activation triggers the assembly of NLRP3 inflammasome^27^. The adapter molecule (ASC) for NLRP3 inflammasome, NLRP3 protein assembly and caspase-1^28^ were also increased in the rat tendons following injury in the RC, suggesting that the amplified inflammation associated with shoulder tendinopathies is an outcome of NLRP3 inflammasome. Also, the HMGB1-RAGE axis contributes to inflammasome activation independent of TLR pathway in the lung tissues^29^, and similar mechanism could be possible to aggravate the inflammatory events associated with RCTI, which warrants further research. None-the-less, these findings suggest that HMGB1 could act as a priming signal for the upregulation, assembly and activation of NLRP3 inflammasome and subsequent IL-1β secretion. The immune cells, including neutrophils and macrophages, are the major sources for IL-1β secretion^30,31^, however, under pathological conditions tenocytes also secrete IL-1β^32^. IL-1β sustains the RCTI pathology by enhancing collagen matrix degradation by the activation of MMPs which results in the persistence of ECM disorganization, inflammation and impaired healing^16^. Moreover, HMGB1 can activate inflammasomes other than NLRP3^27^. However, more research is warranted to validate these aspects in RCTI. Recently, we reported the expression of HMGB1 and RAGE in the tenocytes of human biceps tendons from RCTI subjects^8^. Also, there are evidence for the existence of TLR4 and TLR2 signaling in tenocytes of various tendon tissues^33^. However, activation of TLRs by tenocytes in inflammatory tendinopathies is still in debate^34^. Our results revealed a drastic increase in TLR4- and TLR2-positive cells (Fig. 2) and immune cells following the RCTI (Fig. 1A and B). The presence of biomarkers like CD68, CD16, and scleraxis/tenomodulin revealed the presence of macrophages, neutrophils and tenocytes, respectively, in the RC tendon tissues following the RCTI in the rat model. Macrophages were predominantly present compared to neutrophils (Supplementary Fig. 2A and B). Both cell types express TLR2 and TLR4^35,36^. The presence of CD16 + neutrophils and CD68 + macrophages in the RCTI tendons in our study suggests that the activation of receptors TLR2/4, RAGE and the TREM1 occurs via HMGB1 and other DAMPs. Moreover, other cell phenotypes like tenocytes, fibroblasts and smooth muscle cells, were found to express these receptors^8^. The main focus of this study was to examine the expression of the mediators of NLRP3 inflammasome pathway in the RC tendons following RCTI. However, the molecular mechanisms underlying the activation and regulation of NLRP3 pathway and the associated cell phenotypes warrant further investigation. None-the-less, these findings suggest that HMGB1 triggers inflammasome activation in immune as well as non-immune cell phenotypes and their cumulative effects sustain inflammation in RCTI^8,37^.

RAGE signaling generates mitochondrial and cytosolic ROS and subsequent oxidative stress and apoptosis. It is now widely accepted that ROS generation by RAGE is mediated by its binding with advanced glycation end products (AGEs) and the activation of downstream signaling which generates mitochondrial and cytosolic superoxide, followed by increased mitochondrial permeability and complex-1 insufficiency^38,39^. Oxidative stress in the tendons causes tendon weakness and RCTI by promoting the apoptosis of tenocytes. The upregulation of MMPs, and the inflammatory mediators like cyclooxygenase-2 (COX-2), and prostaglandin E_2_ were found to be associated with altered collagen phenotypes in RC tendon tear^40^. The RAGE expression was found to be higher in Group-2 tendons of our study which is consistent with increased tendon damage when compared with other two groups (Fig. 3A, B). Hence, the RAGE signaling can induce tendon damage by generating ROS apart from its role in NLRP3-mediated pathology.

The danger-associated molecular pattern (DAMP), HMGB1, has been identified to be a ligand for NLRP3 inflammasome activation where the cell surface proteins, including RAGE, TLR2/4 and TREM-1, were identified to be the potent receptors^8,13^. The recent reports revealed that activated immune cells and necrotic cells are the major sources of HMGB1. However, being a hypovascular tissue we speculate that the HMGB1 might be released from damaged tenocytes and/or tenoblasts which warrants confirmation in the rat tendon. The released HMGB1 activates canonical inflammasome via the activation of Caspase-1 and non-canonical inflammasome through Caspase-11. The maturation of proIL-1β and pro-IL-18 by Caspase-1 requires a direct physical interaction of Caspase-11 and Caspase-1. However, HMGB1 can bypass Caspase-1 signaling and can induce pyroptosis mediated through Caspase-11 even in the absence of Caspase-1^41^. Even though more than 121 substrates have been identified, Caspase-1 is not considered to be a typical regulator of apoptosis. Instead, Caspase-1 is a potent inducer of pyroptosis which acts by the proteolytic activation of proIL-1β to IL-1β^42^. These findings signify the multiple modes of HMGB1 signaling in aggravating RCTI. Moreover, the individual/synergistic effects of other DAMP molecules, such as HSP-70 and uric acid, warrants further investigation^43^.

Mitochondrial dysfunction in tenocytes has been considered to be a risk factor for the development of RCTI and the role of mitochondria in tendon regeneration is still in debate^44^. Recent reports revealed that the transfer of mitochondria from healthy and regenerative cells like mesenchymal stem cells (MSCs) through ‘tunneling nanotubes’ (TNTs) to the diseased somatic cells could be a possible regenerative mechanism^45,46^. In contrast, there are reports that the mitochondria of the damaged cells act as DAMPs and aggravate the pathology^47^. Moreover, elevated mitochondrial biogenesis associates with inflammation and ischemia in pathologic and regenerative tissues^48^. However, the impact of mitochondrial biogenesis in activating inflammation and promoting the healing is controversial^49^. Interestingly, recent studies found out that increased mitochondrial biogenesis associates with the regeneration of skeletal muscle tissue^50,51^. However, the status of mitochondria in RCTI is unknown. The RCTI tendons of Group-1 and Group-2 in our studies showed a significant upregulation of the mitochondrial biogenesis marker PGC-1α which was decreased in Group-3 (Fig. 6C and D). Based on these findings, it is reasonable to speculate that mitochondrial activity is required either for eliciting inflammatory responses or for triggering healing responses in rat shoulder tendons. Of course, further investigations are warranted.

ECM disorganization, following the RC tendon injury, has been considered to be major pathological event associated with poor functional outcome of tendon. RC tendon of human and animal models exhibits ECM disorganization and collagen fibers disruption, resulting in mechanical weakness^52,53^. Type-1 collagen (COL1) forms the major component of tendon matrisome and we recently reported the decrease in COL1 with an increase in COL3 in the biceps tendons of RCTI patients. The alteration in COL1:COL3 ratio has been attributed to the elevated activities of various MMPs^54^. Interestingly, the restoration of COL1:COL3 was observed during the course of healing response in the tendon tissues of RCTI rats (Fig. 7A and B). Interestingly, this increase in COL1:COL3 ratio in Group-2 and Group-3 RCTI tendons corresponded with the decreased expression of NLRP3 mediators, which suggests a correlation between ECM disorganization and NLRP3 pathway. In the bone tissue, bone matrix degradation components trigger NLRP3 pathway^55^. In this context, we speculate that the degradation fragments of tendon matrix could act as DAMPs to trigger NLRP3 inflammasome pathway. However, the tendon matrix-DAMP-NLRP3 axis warrants further research.

Triggering receptors expressed on myeloid cell-1 (TREM-1), the member of Ig superfamily of receptors, activates inflammation by amplifying TLRs and NLRs and downstream NLRP3 signaling. TREM-1 biology in inflammation is an emerging field however, the underlying mechanistic events remain to be further investigated^56^. Our recent article describes the upregulation of HMGB1, RAGE and TREM-1 in the biceps tendons of human RCTI patients and tenocytes are capable of secreting TREM-1 along with neutrophils and macrophages^8^. Previous reports showed that TREM-1 activation results in IL-1β release in immune cells like monocytes suggesting its link to NLRP3 signaling^57^. Also, TREM-1 elicits its pro-inflammatory effects through its adaptor DAP12 and downstream NF-κB activation^58^. The stimulation of intracellular Ca^2+^ mobilization following TREM-1 activation triggers mitochondrial Ca^2+^ overload and ROS production^59^. ROS is another potent activator of NLRP3 inflammasome^60^. Along with TLRs and RAGE, HMGB1 also act as a ligand for TREM-1 and the upregulation of these mediators following RCTI suggests the cumulative effect of these mediators in the pathogenesis of RC tendon inflammation.

Pharmacological inhibition of NLRP3 prevents the fat accumulation in hepatocytes which suggests the potential role of NLRP3 in promoting fatty infiltration in tissues under pathological conditions^61^. In concert with TLR4, TREM-1 amplifies the fatty infiltration by enhancing cellular lipid uptake and transport in myeloid cells^62^. Similarly, the upregulation of HMGB1 is closely associated with the fat deposition in hepatocytes. This suggests that the priming of HMGB1 with TLR2/4, TREM-1 and/or RAGE accelerate fatty infiltration. However, no such reports are available in the tendon tissue. Furthermore, the fatty acids are the key activators of inflammasomes, and these effects are mediated through mitochondrial ROS, AMP-activated protein kinase (AMPK) and Unc-51–like kinase-1 (ULK1) autophagy signaling which ends up in IL-1β upregulation^63^. So, the fatty infiltration in RC tendons could also correlate with the activation of NLRP3 inflammasome. Moreover, the persistence of fatty infiltration in RCTI tendons has been reported to delay the healing responses by sustaining the inflammatory responses^37^. However, literature regarding the association of RCTI-fatty infiltration and NLRP3 inflammasome are unavailable which warrants more research. Furthermore, the direct involvement of HMGB1 in RCTI-fatty infiltration is yet to be examined.

Priming of TLRs and RAGE by HMGB1 cause NF-κB signaling to upregulate the cellular expression of NLRP3 and subsequent stabilization by deubiquitination. However, ubiquitination in a linear fashion is required for ASC binding to NLRP3 assembly. Interaction with NLRP3 converts ASC to form a prion-like conformation which results in long ASC filaments that are perquisite for NLRP3 inflammasome activation. Then, the procaspase-1 binds to ASC and forms another prion-like filament which branches off from ASC filaments. This brings procaspase-1 units in close proximity and induces the autoproteolytic activation of procaspase-1 to caspase-1 and subsequent activation of pro-IL-1β to active IL-1β^64^. In addition, NLRP3 inflammasome activation has been linked to multiple cellular signaling events like K^+^ efflux, Ca^2+^ signaling, mitochondrial dysfunction, and lysosomal leakage^65^, and these processes still remain to be investigated in RCTI. Interestingly, all the key molecular players, including HMGB1, RAGE, TLR4, TLR2, ASC, NLRP3, caspase-1 and IL-1β, were found to be upregulated in the tendon tissues of RCTI rats and were progressively downregulated during the course of healing. These data strongly suggest that the inflammation associated with RCTI results from NLRP3 inflammasome activation where HMGB1 acts as the major priming signal.

Even though DAMPs, such as HMGB1 and S100A9, and the receptors, TLR2/4, TREM-1 and RAGE, have been found to be associated with RCTI,^8,66^ this is the first report on the NLRP3 inflammasome activation and associated inflammatory pathway in RCTI. Our findings could suggest several therapeutic targets/approaches to inhibit NLRP3 assembly; these include the prevention of HMGB1 thiolation at Cys-106 and Cys-23 and Cys-45 disulfide formation, inhibition of TLRs and RAGE, prevention of ASC and procaspase-1 filament formation by targeting deubiquitination, and inhibition of final product IL-1β. The translation of these findings to clinical arena would open opportunities to develop novel therapeutic strategies for the management of RCTI, however warrants more research.

The potential limitations of our study include the difficulty to identify muscle-tendon interface after RCTI, presence of suture in the RC tendon caused the loss of tissue during processing and staining, and active healing responses due to young age of rats. In addition, the small size and limited amount of tendon tissue (especially the control tendons) from rat shoulder did not allow us to perform mRNA transcriptomic analysis or proteomic analysis by Western blot. Also, the quadruped rat model of the rotator cuff to mimic human RCTI might not be appropriate. However, the basic anatomy and many of the pathophysiological features of the rat shoulder could be considered similar to that in human^67^. But, the pathological features of human RC tendons, such as post-injury compression, exposure to synovium and surgical repair, might not be fully represented in rats. Nonetheless, the novel findings warrant further research to translate these findings to better therapeutic intervention in human subjects.

### Methodology

#### Animals

The experimental animal protocol was approved by the Institutional Animal Care and Use Committee of Creighton University. All methods in the animal care and procedures in this research protocol were performed in accordance with the NIH and OLAW guidelines. Twenty-one male Sprague Dawley rats of age 8–10 weeks and weight ranging from 230–250 g were recruited for the studies. The animals were acclimatized for 7 days in 12/12 light-dark cycle and were provided with uninterrupted access to food and drinking water throughout the study.

#### Surgically-induced RCTI rat model

The rats were divided into 3 groups → 3–5 days post-injury (Group-1), 10–12 days post-injury (Group-2), and 22–24 days post-injury (Group-3). Each group consisted of seven rats. All surgical procedures were performed under sterile conditions. Hairs at the incision site were removed, the skin was cleaned using a skin disinfectant and rinsed with ethanol prior to surgery. The animals were then anesthetized with 80 mg/Kg ketamine (IP) and 5 mg/Kg xylazine (IM), and 2% isofluorane was used for maintaining anesthesia during surgery^8^. The anesthetized animals were positioned in the right lateral position where the left forelimb including the shoulder was scrubbed again with antiseptic solution and was draped using sterile drapes. A skin incision was made over lateral aspect of the shoulder over the acromion. The deltoid muscle was sharply detached from the postero-lateral aspect of the acromion, and the acromion was then retracted. The rotator cuff tendons were exposed, and a suture was placed through each of the supraspinatus and infraspinatus at the musculo-tendon junction to control the tendon stumps after detachment. The supraspinatus and infraspinatus tendons were then sharply detached from the greater tuberosity using a No. 11 surgical blade. After detachment, approximately 1.5 mm of the tendon stump from lateral side was resected further in order to prevent the healing of the tendon stump back to the greater tuberosity. The tendon was transected at the tendon bone tendon interface and allowed to retract. The retracted tendon stump was then marked with a non-absorbable suture so that the tendon stump could be identified at sacrifice. Approximately 4 mm of retraction was observed in all specimens. The wound was then irrigated with sterile saline and closed using sterile metallic skin staples.

The animals were then allowed to live normally in the cage to evaluate the extent of RCTI and repair responses. The RC tendons from the contralateral side were used as control. CO_2_ euthanasia was employed to sacrifice the animals. The animals of Group-1, Group-2 and Group-3 were sacrificed after 3–5 days, 10–12 days and 22–24 days, respectively.

The animals were recovered from anesthesia after 30–45 min post-surgically and started cage activities. Pain medication was administered, and no symptoms of pain or distress were exhibited by the rats. SC injection of 0.01 mg/kg Buprenorphine was given post-operatively to the RCTI rats twice a day for 2 days. The first dose of Buprenorphine was administered after the surgery but before the rats recovered from anesthesia. After two days acetaminophen was given orally (~30 mg/day) through the drinking water for 5 days. The animals started using their injured limb for movements normally after 24 h post-surgery. No mortality was encountered during anesthesia, surgery or post-surgery. The animals were sacrificed at the defined time periods for RC tissues harvest. After sacrifice, the tendon tissue was dissected from bone and muscle using scalpel blade (#15) using a binocular surgical Loup. Care was taken to avoid bone and muscle in the harvested tissue.

The harvested tendon tissues were assessed for its macroscopic anatomy for neo-fibrous tissue formation. Tissues were then fixed in formalin, paraffin embedded and sections of 5μm thickness were taken onto microscopic slides for further analyses.

### Histology

The deparaffinized sections were used for H&E and pentachrome staining to examine the tissue morphology and ECM organization using our previously reported protocol^33^. After staining, the slides were imaged using an inverted microscope attached with an imaging camera (Olympus BX51; Olympus America, Center Valley, PA) after mounting with xylene based mounting media. Qualitative histology examination was performed on H&E images. However, the parameters of Bonar’s and Movin’s scoring scale, including the appearance of tenocytes, collagen organization, vascularity, and cellular density, were taken into consideration for the microscopic evaluation of the harvested tendon. Video was also captured for the RCTI tendons to provide a high field view of the pathology.

### Immunofluorescence

The tissue sections were analyzed for protein expression using immuno-double staining following the standard published protocols^68,69^. The sections were heated at 95°C for 20 min using HIER buffer (Heat Induced Antigen Retrieval) for antigen retrieval and then blocking solution (0.25% Triton X-100 and 5% horse serum in PBS) added and kept at room temperature for 2 hrs. The primary antibodies were purchased from Santa Cruz Biotechnology (1:50 dilution) and Abcam (1:200 dilution) and were TLR-4 (sc-293072), HMGB-1 (sc-56698), RAGE (sc-365154), ASC (sc-271054), IL-1β (ab9722), PGC-1α (sc-518025), Collagen 1 (ab90395), Collagen 3 (ab7778), Caspase-1 (ab1872), NLRP3 (ab210491), and TLR2 (ab191458). Fluorochrome-conjugated secondary antibodies, donkey anti-mouse, donkey anti-goat and goat anti-rabbit with a dilution of 1:200 were used for detecting the primary antibodies. The secondary antibodies used were alexafluor-488 (green) or alexafluor-594 (red), same for each group and corresponding contralateral controls and the color of fluorochrome of secondary antibodies had no interference on the analysis since the comparisons were made between the contralateral controls. Nuclei were counterstained with 4′,6-diamidino-2-phenylindole (DAPI) and imaged using a fluorescent microscope (Olympus BX51; Olympus America, Center Valley, PA) and the mean fluorescence intensity (MFI) was quantified using ImageJ software. MFI values corresponding to the each RCTI groups were compared with the contralateral controls of the same group. A negative control with secondary antibodies alone was also treated in a similar manner to minimize the auto exposure. Four DAPI images of 40x magnification from different specimen from each RCTI groups and control were randomly examined for cell count using ImageJ.

### Statistical analysis

The results of immunofluorescence intensity have been expressed as mean ± SEM and the statistical significance was evaluated by one-way ANOVA using GraphPad Prism software and the level of significance was set at *p *<* 0.05*. For intensity calculation, images were taken randomly from different fields of each specimen on different slides. Depending on the size of tendon and the adherence of the tissue specimen on the slides (in some cases tissue section did not adhere due to the presence of sutures), 1–2 slides from controls and 2–3 slides from RCTI groups were analyzed. The average intensity of images quantified from each individual specimen was considered to be the MFI for the expression of the specimen. The average values of all individual specimens from each group were utilized to calculate average mean and SEM to perform statistical analysis. The RCTI groups were compared with the contralateral controls in the same experimental group.

## Conclusion

**Figure 8:**
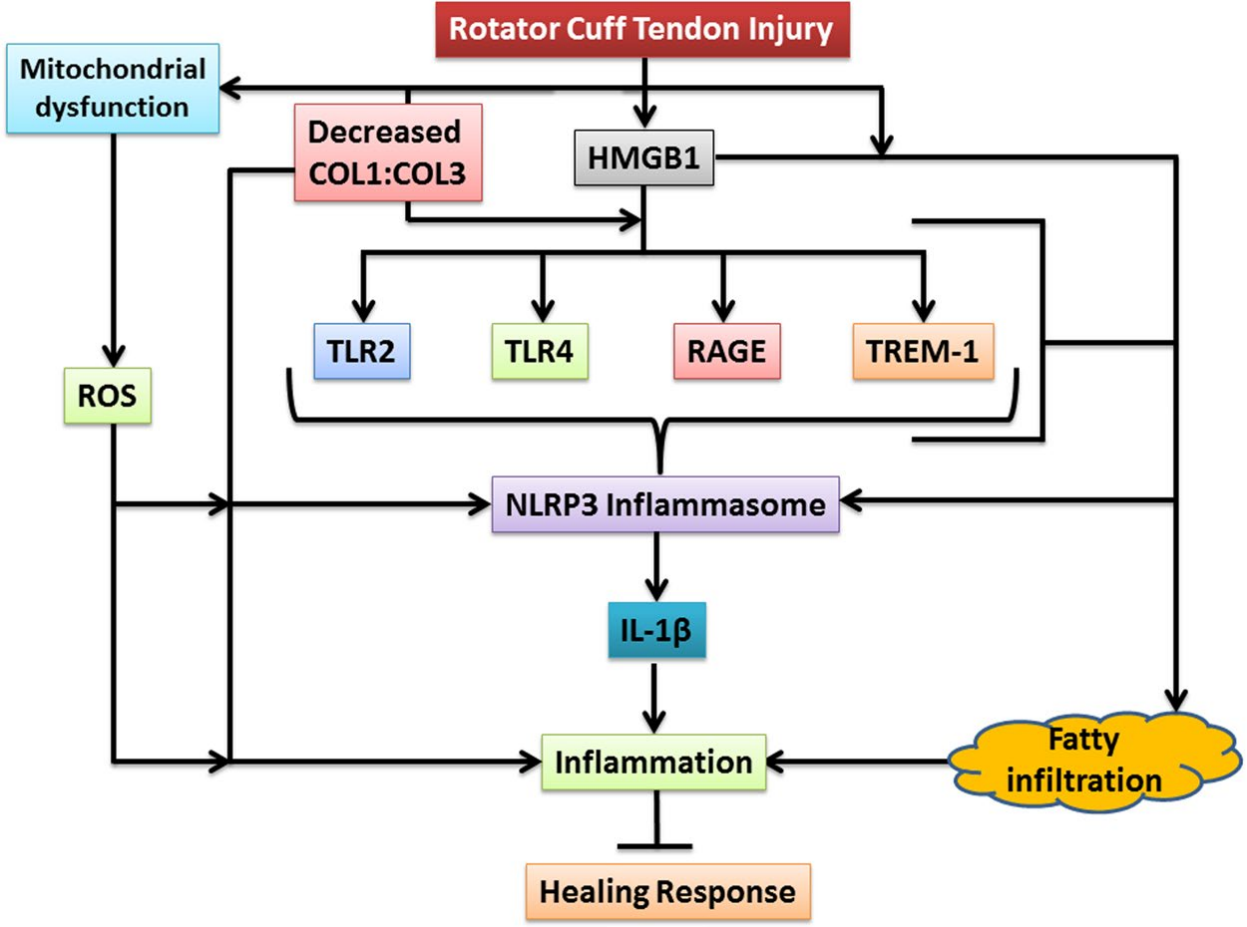
The proposed mechanism of molecular interplay associated RCTI: The rotator cuff tendons following injury undergo hypoxic stress which aggravates the pathology. The injured cells undergoing apoptosis or necrosis release HMGB1 that triggers a battery of receptors including TLRs, TREM-1, and RAGE. The downstream signals and the cellular response from the activated receptors converge to trigger NLRP3 inflammasome signaling resulting in IL-1β release and subsequent inflammation. Also, HMGB1 accelerates fatty infiltration via RAGE and TREM-1 and the fatty infiltrated tendon tissue is highly susceptible for inflammation. Moreover, following the RCTI, tendon matrix homeostasis is disturbed resulting in a decrease in COL1:COL3 ratio where the matrix degradation fragments are believed to act as DAMPs to activate NLRP3. The mitochondrial dysfunction following the tendon injury increases the ROS level which is another trigger for NLRP3 inflammasome and inflammation. The cumulative effects of these mediators, primed by HMGB1, result in the delayed healing responses.

The surgically-induced RCTI rat model displayed pathological features as revealed by anatomical and histomorphological alterations comparable to the classical symptoms associated with human RCTI. The molecular mediators associated with NLRP3 inflammasome were upregulated with respect to pathology and declined during the course of healing. The prime signal for these events appears to be HMGB1 which can activate multiple pathways through the receptors including TREM-1, TLR4, TLR2 and RAGE, which ultimately triggers NLRP3 inflammasome assembly and activation to release IL-1β to induce increased ECM disorganization and inflammatory response following injury in the rotator cuff tendon resulting in the inhibition of healing response (Fig. 8). Further investigation of NLRP3 inflammasome signaling in RCTI could identify target(s) to develop novel therapeutic strategies in the management of RCTI.

## Electronic supplementary material

Supplementary Figures

Supplementary Video-1

Supplementary Video-2
