# Supplementary material for: Association of Inflammatory Responses and ECM Disorganization with HMGB1 Upregulation and NLRP3 Inflammasome Activation in the Injured Rotator Cuff Tendon
Source: Sci Rep. 2018 Jun 11;8:8918. doi: 10.1038/s41598-018-27250-2 (PMC5995925; doi:10.1038/s41598-018-27250-2)
Supplement: Supplementary file 1 — Supplementary Figures [file 41598_2018_27250_MOESM1_ESM.docx]

**Supplementary Data**

**Title of the Manuscript**: Association of Inflammatory Responses and ECM Disorganization with HMGB1 Upregulation and NLRP3 Inflammasome Activation in the Injured Rotator Cuff Tendon

**Authors**: Finosh G Thankam, Zachary K Roesch, Matthew F Dilisio, Mohamed M. Radwan, Anuradha Kovilam, R. Michael Gross, and Devendra K. Agrawal

**Manuscript #**: SREP-18-06939


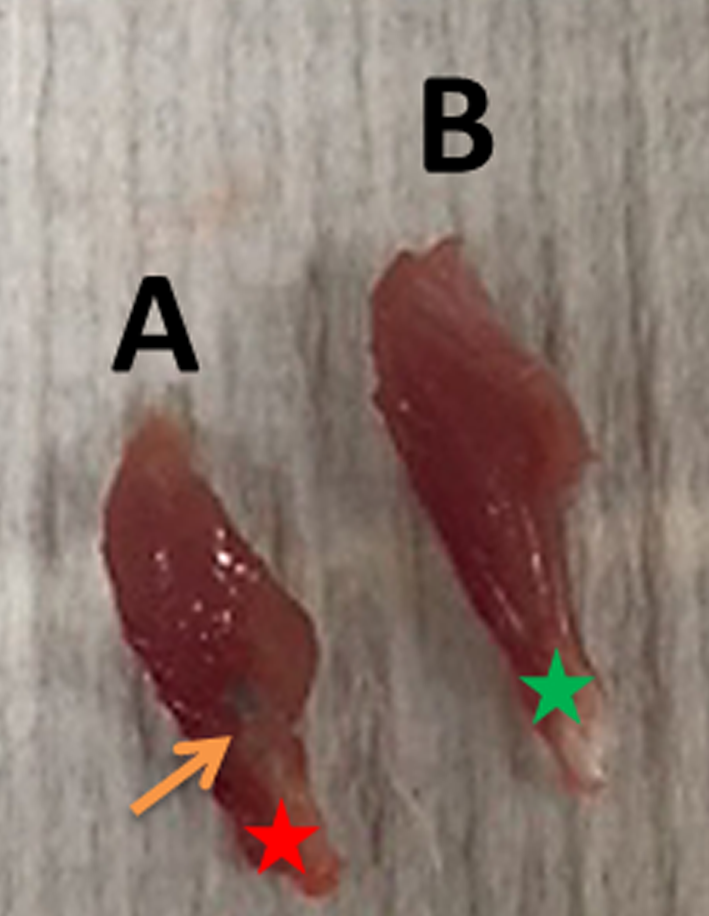
 **Supplementary Figure 1**: Anatomical appearance of RC tendon of RCTI rats (A) when compared with control (B). The green star points the normal tendon which was white shiny appearance while the red star indicates the neo-tendon tissue formed after 22 days of injury which appeared less shiny and distorted. The orange arrow shows the suture used for tendon transection.


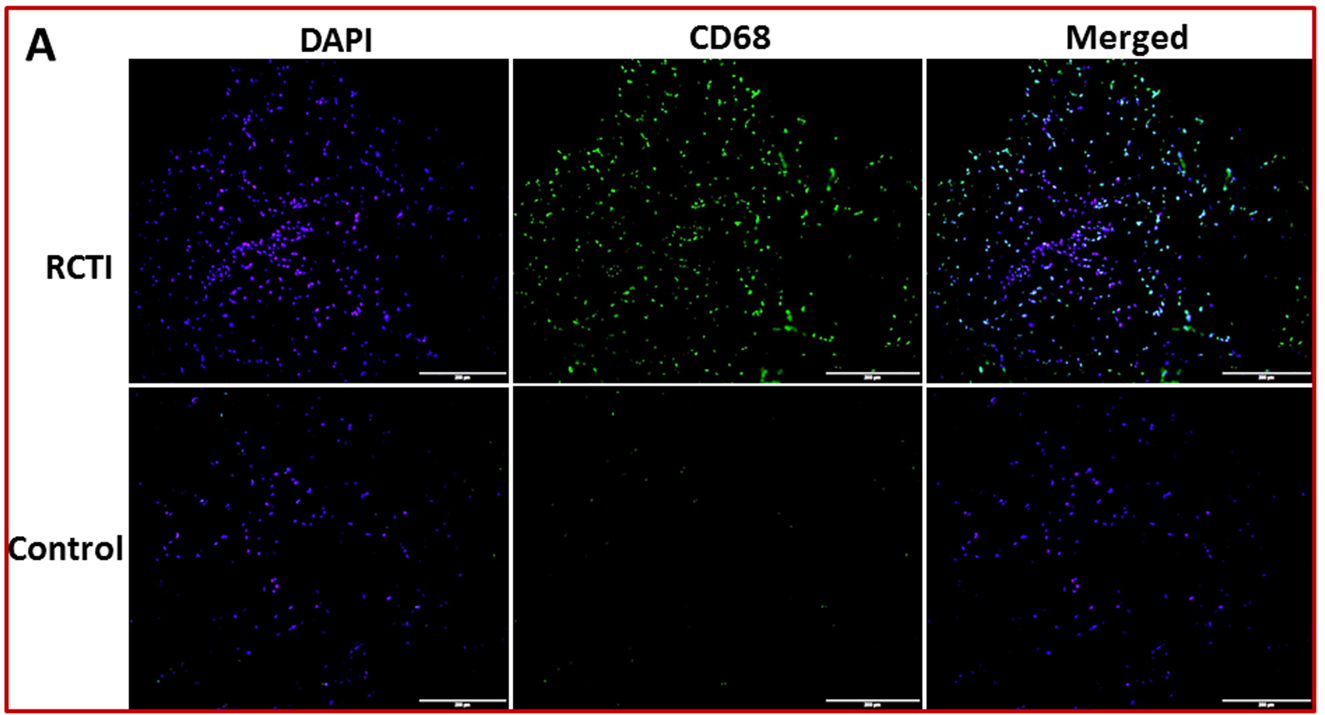


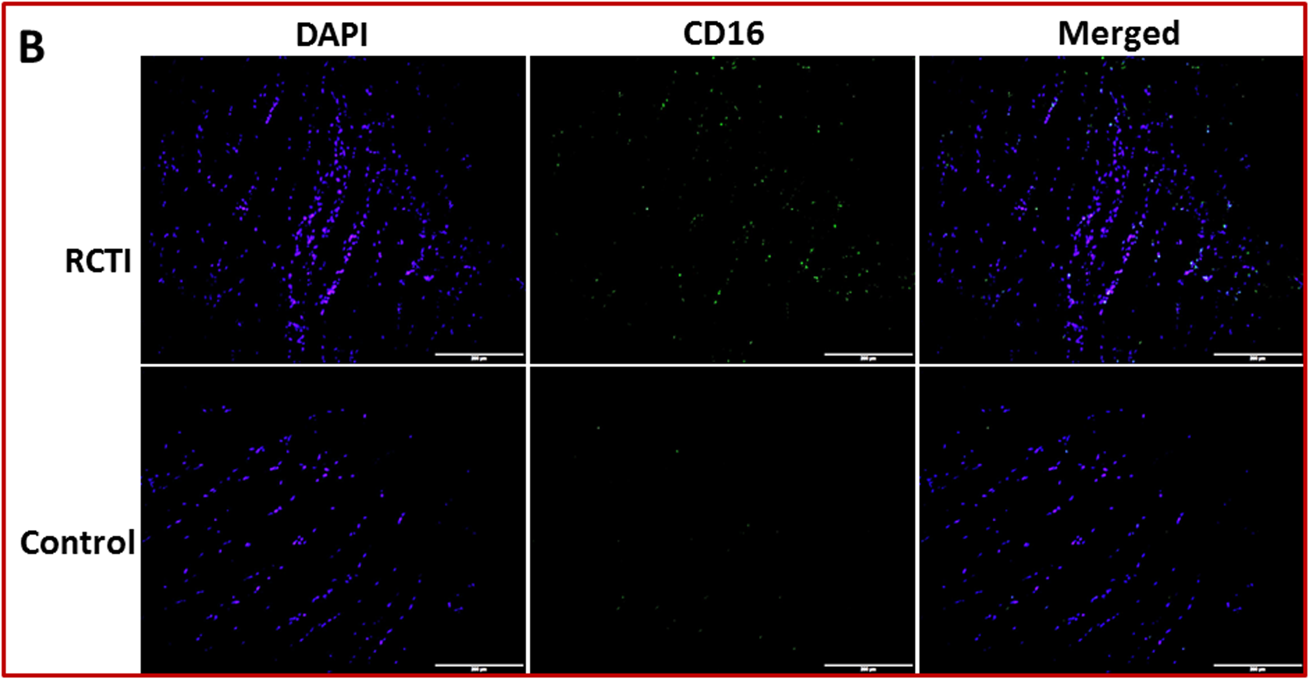


**Supplementary Figure 2**: Representative images for the immunofluorescence analysis for the expression of (A) CD68+ macrophages and (B) CD16+ neutrophils showing increased expression in RCTI tendon in comparison to the control. The images show more macrophages than neutrophils in RCTI tendons. Images were acquired at 20x magnification using CCD camera attached to the Olympus microscope.
